# Biventricular Myocardial Fibrosis and Sudden Death in Patients With Brugada Syndrome

**DOI:** 10.1016/j.jacc.2021.08.010

**Published:** 2021-10-12

**Authors:** Chris Miles, Angeliki Asimaki, Irina Chis Ster, Michael Papadakis, Belinda Gray, Joseph Westaby, Gherardo Finocchiaro, Carlos Bueno-Beti, Bode Ensam, Joyee Basu, Gemma Parry-Williams, Hamish MacLachlan, Khari A. Edwards, David Johnson, Maite Tome, Sanjay Sharma, Mary N. Sheppard, Elijah R. Behr

**Affiliations:** a Cardiovascular Clinical Academic Group, St George’s University Hospitals’ NHS Foundation Trust and Molecular and Clinical Sciences Institute, St George’s, University of London, London, United Kingdom; b Institute of Infection and Immunity, St George’s University of London, London, United Kingdom; c Department of Cardiology, Royal Prince Alfred Hospital, Sydney, New South Wales, Australia; d Faculty of Medicine and Health, University of Sydney, Sydney, New South Wales, Australia; e Guy’s and St Thomas’s Hospital, London, United Kingdom

**Keywords:** Brugada syndrome (BrS), collagen, myocardial fibrosis, sudden arrhythmic death syndrome (SADS), ACMG, American College of Genetics and Genomics, BrS, Brugada syndrome, CMR, cardiac magnetic resonance imaging, CRY-CCP, Cardiac Risk in the Young Centre for Cardiac Pathology, IVS, interventricular septum, LV, left ventricular, RV, right ventricular, RVOT, right ventricular outflow tract, SADS, sudden arrhythmic death syndrome, SCD, sudden cardiac death

## Abstract

**Background:**

Electrophysiological, imaging, and pathological studies have reported the presence of subtle structural abnormalities in hearts from patients with Brugada syndrome (BrS). However, data concerning disease involvement outside of the right ventricular outflow tract are limited.

**Objectives:**

This study sought to characterize the presence and distribution of ventricular myocardial fibrosis in a cohort of decedents experiencing sudden cardiac death caused by BrS.

**Methods:**

The authors evaluated 28 whole hearts from consecutive sudden cardiac death cases attributed to BrS and 29 hearts from a comparator group comprised of noncardiac deaths (control subjects). Cardiac tissue from 6 regions across the right and left ventricle were stained with Picrosirius red for collagen and tissue composition was determined using image analysis software. Postmortem genetic testing was performed in cases with DNA retained for analysis.

**Results:**

Of 28 BrS decedents (75% men; median age of death 25 years), death occurred in sleep or at rest in 24 of 28 (86%). The highest proportion of collagen was observed in the epicardial right ventricular outflow tract of the BrS group (23.7%; 95% CI: 20.8%-26.9%). Ventricular myocardium from BrS decedents demonstrated a higher proportion of collagen compared with control subjects (ratio 1.45; 95% CI: 1.22-1.71; *P <* 0.001), with no significant interactions with respect to sampling location or tissue layer. There was insufficient evidence to support differences in collagen proportion in *SCN5A*-positive cases (n = 5) when compared with control subjects (ratio 1.23; 95% CI: 0.75-1.43; *P =* 0.27).

**Conclusions:**

Brugada syndrome is associated with increased collagen content throughout right and left ventricular myocardium, irrespective of sampling location or myocardial layer.

Brugada syndrome (BrS) is an inherited arrhythmic disorder diagnosed in the presence of a characteristic type 1 ECG pattern employing consensus-based criteria (1,2). Since the first electrocardiographic descriptions of BrS (3,4), its pathophysiological basis remains unresolved. Several pathological studies report a wide variety of abnormal cardiac tissue architecture in patients with BrS, including those harboring pathogenic *SCN5A* variants. Histological findings of interstitial fibrosis, inflammatory infiltrates, and fibrofatty infiltration have been described, suggesting pathological overlap with cardiomyopathic and inflammatory disorders including arrhythmogenic cardiomyopathy and myocarditis (5, 6, 7). The presence of structural abnormalities in BrS are corroborated by abnormal electrical activation and low voltage areas during electrophysiological study, where fractionated electrocardiograms and conduction delay appear localized to right ventricular (RV) epicardium (8,9).

Although usually considered a disease exclusive to the RV, histopathological evaluation of a small series of autopsied sudden cardiac death (SCD) decedents with familial evidence for BrS had suggested increased collagen content within left ventricular (LV) as well as RV myocardium (10). Some patients with BrS also exhibit LV midwall late gadolinium enhancement on cardiac magnetic resonance imaging (CMR) (11). However, whether BrS results in pathological abnormalities outside of the RV has yet to be fully elucidated. In this study, we sought to characterize the pathological characteristics of the largest reported cohort of BrS decedents and SCD cases with familial BrS to date, testing the hypothesis that BrS is associated with fibrosis within both right and LV myocardium. Using automated digital pathology software, we performed blinded quantification of histological cardiac tissue composition and collagen content in a clinically validated BrS SCD cohort in comparison to noncardiac death control subjects.

## Methods

### Background

In the United Kingdom, all unexpected sudden deaths are referred to the coroner and an autopsy is performed. The Cardiac Risk in the Young Centre for Cardiac Pathology (CRY-CCP) at St George’s, University of London, provides an expert national cardiac pathology service. Referral to the CRY-CCP is initiated voluntarily by the coroner’s pathologist following an unexplained sudden death, or if there is suspicion of an inherited heart condition. The center receives whole hearts or cardiac tissue blocks from >500 referrals of sudden death each year. Demographics, clinical data, and toxicological and pathological findings are entered prospectively into a database. Ethical approval for this study was granted by the London Stanmore National Health Service Research Ethics Committee (reference: 10/H0724/38).

### Study setting and cohorts

**Figure 1:**
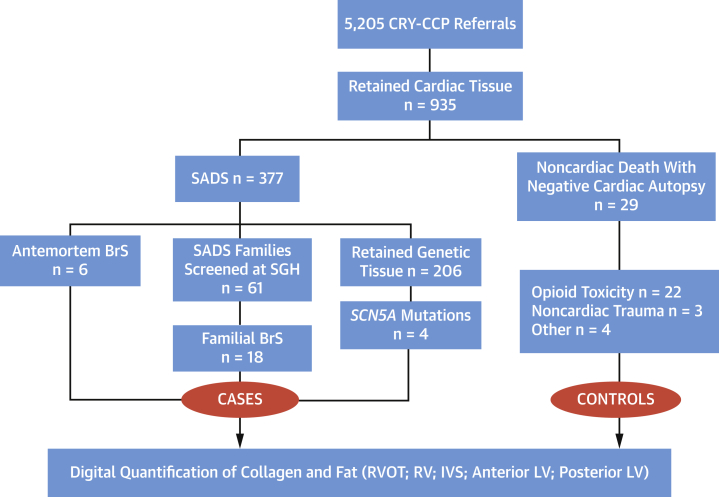
Selection of the Study Population Flow chart depicting the total number of SCD referrals to the CRY-CCP and selection of BrS cases and noncardiac control subjects with retained cardiac tissue. BrS = Brugada syndrome; CRY-CCP = Cardiac Risk in the Young Centre for Cardiac Pathology; IVS = interventricular septum; LV = left ventricle; RV = right ventricle; RVOT = right ventricular outflow tract; SADS = sudden arrhythmic death syndrome; SCD = sudden cardiac death; SGH = St George's Hospital London.

Between 1994 and 2018, 5,205 cases were referred to the CRY-CCP for specialist cardiac autopsy. A total of 935 cases had retained cardiac tissue suitable for histopathological evaluation. Six cases with an antemortem diagnosis of BrS and 18 sudden arrhythmic death syndrome (SADS) cases with a familial diagnosis of BrS were identified over this time period. Of these, 14 cases (58%) had retained genomic tissue suitable for postmortem genetic testing. In addition, cardiac tissue from 4 SADS cases harboring pathogenic *SCN5A* variants (with expected loss-of-function) were identified from the same base SCD cohort (Table 1). The comparator group (n = 29), referred to as control subjects, comprised of consecutive noncardiac deaths referred to the CRY-CCP, where cardiac tissue was retained following coronial judgment or inquest (Figure 1).

**Table 1:** Demographics, Clinical, and Genetic Characteristics of BrS Decedents

Case ID	Antemortem Dx	Sex	Ethnicity	Age at Death, y	Circumstances of Death	Type 1 ECG	FH BrS	FH Unexplained SCD Age <45 y	Syncope	Shanghai Score	First Degree Relatives Screened, n	*SCN5A* (P/LP/VUS)
BrSA1	BrS	F	White	60	Died at rest	1	0	1	0	7 (proband)	NA	Negative
BrSA2	BrS	M	White	25	Died in sleep	1	0	0	0	6.5 (proband)	NA	Negative
BrSA3	BrS	M	White	44	Died at rest	1	0	0	0	7 (proband)	NA	Pathogenic nonsense c.3944C>G (p.S1315X)
BrSA4	BrS	M	White	25	Died in sleep	1	0	0	0	6.5 (proband)	NA	Negative
BrSA5	BrS	M	Asian	45	Died at rest	1	0	0	0	6.5 (proband)	NA	VUS missense c.50C>T (p.T17I)
BrSA6	BrS	M	White	37	Died at rest	0	1	1	0	6 (proband)	NA	Not performed
BrSF7	None	M	White	31	Died at rest	0	0	0	0	2.5 (mother)	2	Negative
BrSF8	None	F	White	17	Died in sleep	0	0	0	0	3 (mother)	3	Not performed
BrSF9	None	F	White	24	Died in sleep	0	0	0	1	3 (mother)	1	Negative
BrSF10	None	M	White	50	Died immediately after exertion	0	0	0	0	2 (daughter)	3	Negative
BrSF11	None	F	White	25	Died in sleep	0	0	0	0	3 (father)	4	Negative
BrSF12	None	M	White	27	Died at rest	0	0	0	0	2 (mother and father positive ajmalines)	3	Not performed
BrSF13	None	M	White	15	Died in sleep	0	0	0	0	2.5 (father and brother positive ajmalines)	3	Not performed
BrSF14	None	M	White	23	Died immediately after exertion	0	0	0	0	4 (mother and sister positive ajmalines)	3	Not performed
BrSF15	None	M	White	27	Died in sleep	0	0	0	0	3 (mother and brother positive ajmalines)	3	Negative
BrSF16	None	M	White	56	Died in sleep	0	0	0	0	2 (son)	5	Negative
BrSF17	None	M	White	19	Died in sleep	0	0	0	0	3 (father)	5	Negative
BrSF18	None	M	White	40	Died at rest	0	0	0	0	2 (sister)	4	Negative
BrSF19	None	M	White	17	Died in sleep	0	0	0	0	3 (father)	3	Not performed
BrSF20	None	M	White	24	Died in sleep	0	0	0	0	3 (father)	3	Not performed
BrSF21	None	M	White	37	Died in sleep	0	0	0	0	3 (father)	3	Not performed
BrSF22	None	M	White	25	Died during exertion	0	0	0	0	3 (father)	3	Negative
BrSF23	None	M	White	23	Died in sleep	0	0	0	0	3 (father)	4	Not performed
BrSF24	None	M	White	23	Died during exertion	0	0	0	0	2 (father)	2	Not performed
BrSG25	None	F	Asian	27	Died in sleep	0	0	0	0	NA	NA	Likely pathogenic missense c.5038G>A (p.A1680T)
BrSG26	None	F	White	24	Died in sleep	0	0	0	0	NA	NA	Pathogenic missense c.673C>T (p.R225W)
BrSG27	None	M	White	23	Died in sleep	0	0	0	0	NA	NA	Likely pathogenic missense c.3665T>G (p.L1222R)
BrSG28	None	F	White	47	Died at rest	0	0	0	0	NA	NA	Likely pathogenic in-frame c.4850_4852delTCT p.Phe1617 del

BrS = Brugada syndrome; BrSA = Brugada syndrome antemortem; BrSF = Brugada syndrome familial; BrSG = SADS cases harboring pathogenic/likely pathogenic *SCN5A* variants; Dx = diagnosis; FH = family history; NA = not available; P/LP/VUS = pathogenic/likely pathogenic/variant of uncertain significance; SCD = sudden cardiac death.

### Cardiac examination

Whole hearts from BrS and control groups were macroscopically and microscopically normal following examination by expert cardiac pathologists. Histological evaluation was performed at the time of referral with hematoxylin and eosin staining. Heart weight, ventricular chamber diameter, and wall thickness were prospectively recorded. Tissue sections (5-μm thickness) were sampled at the midventricular level or from the anterior aspect of the right ventricular outflow tract (RVOT). In total, 6 cardiac regions from retained tissue sections were evaluated: RVOT; RV; anterior interventricular septum (IVS); posterior IVS; anterior LV; and posterior LV. The RV section included 3 sampled locations from the right anterior, lateral, and posterior walls. Sections were stained with Picrosirius red for collagen, and slides were scanned using 20× magnification on an automated high-resolution scanner (Nanozoomer, Hamamatsu Photonics).

### Clinical data collection

Clinical records from antemortem BrS cases (n = 6) and where SADS families had undergone evaluation at St George’s Hospital London were retrospectively reviewed before study inclusion. Inclusion criteria for SADS cases were as follows: a witnessed sudden death within 1 hour of symptoms, or last seen alive within 12 hours of death; aged 1-64 years; no prior recorded cardiac disease; no history of epilepsy; a normal full coroner’s post mortem; negative toxicology; and a normal expert cardiac pathologists’ examination (12). In total, families of 61 SADS probands with retained cardiac tissue underwent clinical evaluation at St George’s Hospital. This included assessment with 12-lead, signal-averaged, and exercise ECGs; echocardiography; and ambulatory cardiac monitoring. Among families without diagnostic features of an inherited cardiac condition, an ajmaline provocation challenge was performed according to an established protocol (13). Cases with a diagnosis of familial BrS were included in the presence of a spontaneous or drug-induced type 1 Brugada ECG pattern in the standard or high precordial leads in at least 1 first-degree relative. Antemortem BrS and familial BrS were diagnosed based on expert consensus criteria (2). Electrocardiogram criteria for a type 1 Brugada ECG pattern consisted of ST-segment elevation with coved morphology ≥2 mm in ≥1 lead among the right precordial leads.

Demographics and clinical data for SCD and control cohorts was obtained following review of the CRY-CCP database. The consensus-derived Shanghai score was calculated for each decedent with an antemortem diagnosis of BrS, and for familial BrS cases, the relative with the highest score following a diagnosis of BrS (1). SADS cases without familial evaluation data but with pathogenic or likely pathogenic *SCN5A* loss of function variants (as per American College of Medical Genetics and Genomics [ACMG] criteria [14]) were identified from the same base cohort. Noncardiac deaths were determined by information from the referring pathologist’s autopsy and final coronial judgment.

### Postmortem genetic testing

Following extraction of DNA from retained splenic tissue, cardiac gene panel testing was performed using the Illumina TruSight Cardio (174 genes) panel (or a custom Agilent SureSelect with equivalent content). Libraries were prepared according to the manufacturer’s instructions and sequenced on the Illumina platform (NextSeq or HiSeq), as previously described and validated (15,16). In total, 77 inherited arrhythmia- and cardiomyopathy-related genes were analyzed across both capture systems (Supplemental Table 1). Variant annotation was then undertaken in-house using SnpEff version 4.3T (build 2017-11-24) (17), GRCh37.75 (Ensembl), and ANNOVAR (version 2017-07-17) (18). Rare variants were defined as those with a minor allele frequency cut-off <0.01% in the ExAC general population database. Nontruncating variants in *TTN* and synonymous variants not located at splice sites were excluded because of their high frequency and lack of certainty of disease causation. Rare variants were then assessed for pathogenicity according to the ACMG criteria (14).

### Automated image analysis

**Figure 2:**
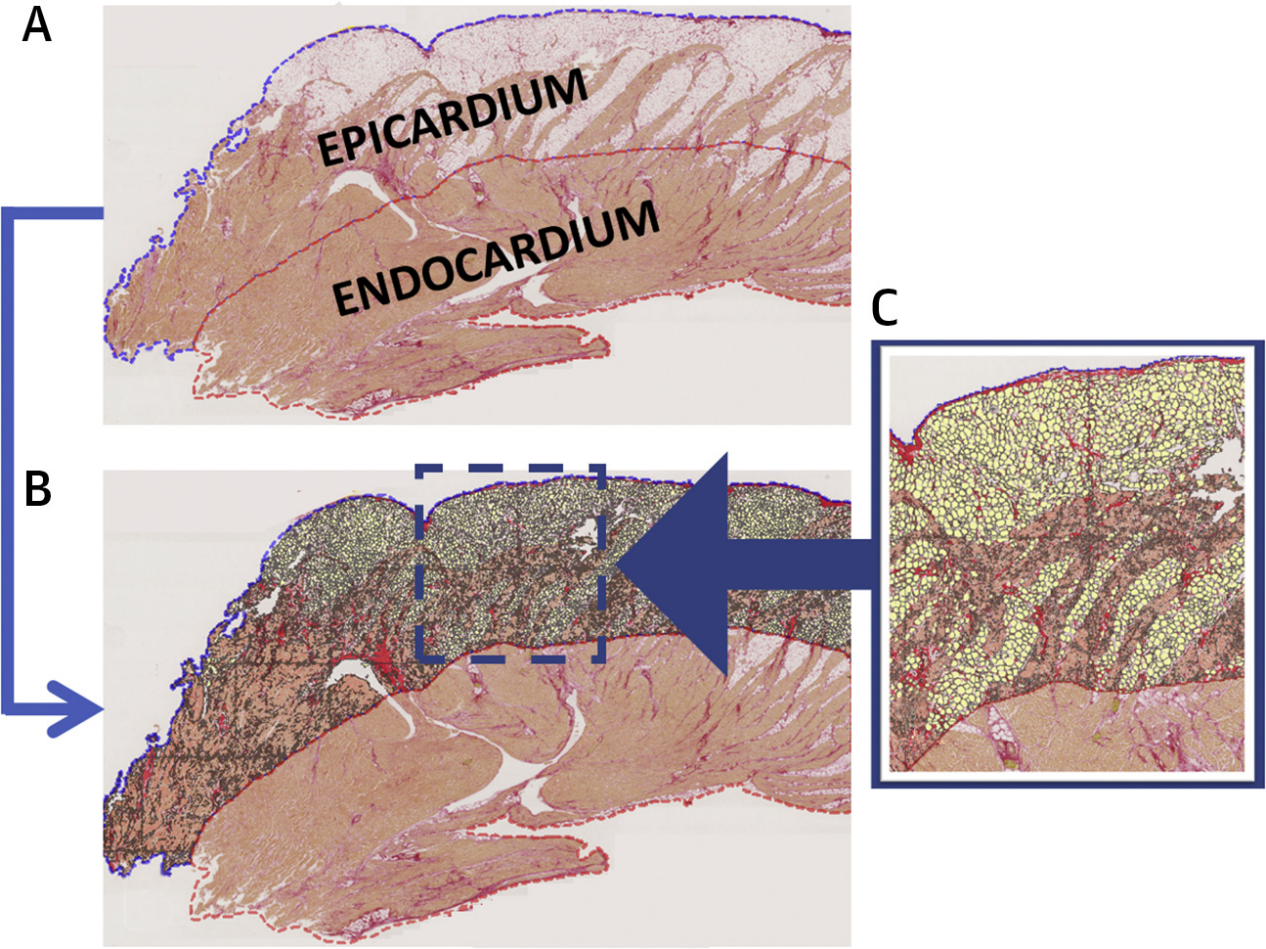
Histological Slide of an RVOT Section From a BrS Decedent **(A)** Automated detection of a digitally transformed histological slide from a RVOT section stained with Picrosirius red to determine epicardial and endocardial layers. **(B)** Digital characterization of epicardial layer tissue composition using Visiopharm software. **(C)** Classification of fat **(yellow)**, collagen **(red)**, and myocytes **(orange)**. Abbreviations as in Figure 1.

Automated calculation of cross-sectional tissue area and quantification of collagen (%), myocytes (%), and fat (%) was performed blinded to case or control group using an application developed within Visiopharm image analysis software (Visiopharm A/S) (Figure 2), as previously reported (19). Proportions were calculated by assessment of collagen and fat area with respect to automated detection of total tissue area. Perivascular collagen surrounding epicardial and intramural vessels was excluded because of high variability in vessel caliber between samples. Exclusion was based on automated lumen detection (elliptical score ≥0.4) incorporating surrounding region of Picrosirius red stain. Total tissue area was categorized into epicardial and endocardial regions (50:50). RV (right anterior, lateral, and posterior sections) and septal (anterior and posterior IVS) tissue areas were combined for the purpose of morphometric quantification.

### Statistical analysis

Continuous normal data are summarized as mean ± SD and skewed data are presented as median (interquartile range [IQR]). Categorical data are presented as groups’ percentages. Differences between group demographics and macroscopic cardiac appearances were compared using a 2-sample independent Student’s *t*-test, Mann-Whitney *U* test, or chi-square (Fisher exact) test, according to their statistical nature and appropriateness of their distributional assumptions. Differences between groups were assessed through regression techniques on outcomes defined by proportions (log transformed) of each tissue component (% collagen, fat, myocytes). These outcomes generated hierarchically dependent data per sampling location (RVOT, RV, IVS, anterior LV, posterior LV) and tissue area (whole block, endocardial layer, epicardial layer), incorporating 2 difference sources of variability. Hence, outcomes were analyzed using multilevel regression techniques for correct inferences. Potential explanatory variables of age at death, sex, and body mass index were included in multivariable analyses. Predictions of collagen and fat proportion at the original scale were expressed in terms of geometric means across groups in the data. Given the exploratory nature of this study, *P* values and 95% CIs presented in this report have not been adjusted for multiple testing. Further investigations are therefore required for subsequent confirmatory phases of the research. Statistical analyses were undertaken using Stata IC/15 (StataCorp). One author (I.C.S.) supervised the data analyses, which were implemented by the first author (C.M.).

## Results

### Brugada SCD population

The majority of the BrS SCD cohort were men (21 of 28; 75%) and white (26 of 28; 93%) with a median age at death of 25 years (IQR: 23-38.5 years). Death occurred in sleep in 16 of 28 (57%) cases and at rest in 8 of 28 (29%) cases. Three deaths (11%) occurred either during physical exertion or immediately after exertion. Prior syncope was documented in 1 (1 of 28; 4%) decedent. Of 6 antemortem BrS cases (6 of 28; 21%), 5 of 6 (83%) had shown a spontaneous type 1 Brugada ECG, 1 later testing positive for a pathogenic *SCN5A* mutation. None of the antemortem BrS group demonstrated features of structural heart disease on echocardiography, and 1 decedent underwent CMR with negative late gadolinium assessment. Among familial BrS cases (18 of 28; 64%), the mean number of clinically evaluated first-degree relatives was 3.2 ± 1.0. Most cases (14 of 18; 78%) were diagnosed based on the presence of a positive ajmaline provocation challenge in 1 first-degree relative, none of whom showed a spontaneous type 1 ECG. The median Shanghai score among antemortem BrS cases was higher than in familial BrS cases (median 6.5 [IQR: 6.5-7] vs 3 [IQR: 2-3], respectively [p = 0.0002]). In SADS cases without familial evaluation or antemortem data, 4 tested positive for pathogenic or likely pathogenic variants in *SCN5A* (presumed to cause loss of function). Deaths in this group similarly occurred at rest or during sleep. None of the 14 BrS cases subjected to postmortem genetic testing demonstrated likely pathogenic or pathogenic variants in non-*SCN5A* arrhythmia and cardiomyopathy-related genes. Demographics as well as clinical and genetic characteristics of the BrS SCD group are presented in Table 1.

### Control group

Most sudden deaths in the noncardiac death control group were caused by opioid toxicity (22 of 29; 76%) or noncardiac trauma (3 of 29; 10%). The majority of control decedents were men (25 of 29; 86%). The mean age at death was 32.1 ± 9.9 (range 18-54) and mean body mass index 28.7 ± 7.3 kg/m^2^. None of the control group had reported antemortem cardiac symptoms or family history of inherited cardiac disease or premature SCD.

### Macroscopic and histological cardiac appearances

Descriptive data and pathological comparisons between case and control subjects are presented in Table 2. There were no significant differences with respect to age at death (*P =* 0.56), sex (*P =* 0.28), or body mass index (*P =* 0.21). Macroscopic cardiac appearances, including height weight and cardiac chamber dimensions, were similar in both case and control subjects. Histological appearances of cardiac tissue from each of the 6 sampled regions demonstrated no evidence of inflammatory infiltrates, fibrofatty infiltration, or overt myocardial fibrosis in either group.

**Table 2:** Comparison of Demographics and Macroscopic Cardiac Appearances Between BrS Decedents and Control Subjects

	BrS (n = 28)	Controls (n = 29)	*P* Value
Age at death, y	30.7 ± 12.1 (25)	32.1 ± 9.9 (33)	0.56
Male	21 (75)	25 (86)	0.28
White	26 (93)	28 (97)	0.53
Weight, kg	80.4 ± 13.1 (80)	88.3 ± 21.2 (83)	0.16
Body mass index, kg/m^2^	26.0 ± 4.2 (25.4)	28.7 ± 7.3 (26.7)	0.21
Heart weight, g	351.5 ± 75.8 (365.5)	359.6 ± 67.1 (351)	0.67
RV chamber diameter	30.3 ± 6.2 (30)	27.9 ± 6.5 (25)	0.17
Anterior RV thickness, mm	2.5 ± 1.1 (2)	2.6 ± 1.0 (2)	0.71
Lateral RV thickness, mm	2.7 ± 1.2 (2.5)	2.7 ± 0.9 (3)	0.83
Posterior RV thickness, mm	3.5 ± 0.9 (3.5)	3.3 ± 0.7 (3)	0.31
RVOT epicardial fat, mm	0.7 ± 1.5 (0)	1.0 ± 1.2 (1)	0.21
RVOT muscle, mm	3.3 ± 1.0 (3)	3.3 ± 0.9 (3)	0.95
LV chamber diameter, mm	33.2 ± 7.2 (35)	31.8 ± 6.1 (31)	0.43
IVS diameter, mm	12.9 ± 2.0 (13.5)	13.9 ± 4.0 (13)	0.45
Anterior LV wall thickness, mm	11.8 ± 2.3 (12)	11.9 ± 2.1 (12)	0.85
Lateral LV wall thickness, mm	12.5 ± 2.2 (12.5)	12.3 ± 2.3 (12)	0.80
Posterior LV wall thickness, mm	11.9 ± 1.8 (12)	11.7 ± 1.8 (11.5)	0.58

Values are mean ± SD (median) or n (%).

BrS = Brugada syndrome; IVS = interventricular septum; LV = left ventricular; RV = right ventricular; RVOT = right ventricular outflow tract.

### Morphometric quantification of collagen and fat

The mean sampled tissue areas for the RVOT, RV, IVS, anterior LV, and posterior LV were 132.5 ± 55.7 mm^2^, 247.6 ± 75.1 mm^2^, 223.5 ± 76.2 mm^2^, 239.5 ± 61.2 mm^2^, and 245.2 ± 56.1 mm^2^, respectively. Table 3 outlines predicted values for the proportion of collagen, fat, and myocytes (total, epicardial, and endocardial) in the RVOT, RV, IVS, anterior LV and posterior LV, stratified by Brugada and control group. As shown in the Central Illustration and Table 3, the highest proportion of collagen was observed in the RVOT epicardium of the BrS group (geometric mean 23.7%; 95% CI: 20.8%-26.9%). Individual-level raw data for collagen proportion are outlined in Supplemental Table 2.

**Table 3:** Predicted Values of Cardiac Tissue Composition Within the RVOT, RV, Septum, and Anterior and Posterior LV

	RVOT		RV		Septum		Anterior LV		Posterior LV	
	Mean	95% CI	Mean	95% CI	Mean	95% CI	Mean	95% CI	Mean	95% CI
Brugada (n = 28)										
Collagen % of tissue area										
Total	21.6	18.8-24.8	21.0	18.3-24.0	12.9	11.4-14.6	12.7	11.0-14.5	14.5	12.6-16.7
Epicardial	23.7	20.8-26.9	23.0	20.3-26.1			12.9	11.3-14.6	12.9	11.3-14.7
Endocardial	19.5	17.0-22.4	18.1	15.8-20.8			12.3	10.7-14.1	15.7	13.7-18.0
Fat % of tissue area										
Total	7.8	5.7-10.6	12.7	9.4-17.2	1.8	1.4-2.4	7.1	5.2-9.6	3.2	2.4-4.4
Epicardial	11.6	8.1-16.5	21.5	15.2-30.5			11.1	7.8-15.7	4.5	3.2-6.5
Endocardial	3.5	2.7-4.5	4.7	3.6-6.0			2.8	2.1-3.6	2.3	1.8-2.9
Myocytes % of tissue area										
Total	62.4	58.6-66.4	60.2	56.5-64.0	82.7	78.4-87.2	76.1	71.5-81.0	80.1	75.3-85.3
Epicardial	50.9	45.0-57.7	45.7	40.5-51.6			67.0	59.2-75.8	79.4	70.2-89.9
Endocardial	73.3	69.9-76.9	73.1	69.7-76.6			83.4	79.5-87.4	79.8	76.1-83.7
Control subjects (n = 29)										
Collagen % of tissue area										
Total	14.6	12.4-17.1	15.9	13.5-16.7	8.6	7.4-9.9	9.1	7.7-10.6	9.9	8.4-10.6
Epicardial	15.4	12.8-18.5	16.3	13.5-19.8			9.4	7.8-11.3	8.8	7.3-10.6
Endocardial	13.3	11.3-15.6	14.0	11.8-16.5			8.7	7.4-10.2	10.4	8.9-12.2
Fat % of tissue area										
Total	10.9	8.0-14.6	14.2	10.5-19.2	1.5	1.2-1.9	7.4	5.5-9.9	3.0	2.2-4.0
Epicardial	16.8	11.7-24.1	24.6	16.8-36.0			12.1	8.4-17.4	4.3	3.0-6.1
Endocardial	4.3	3.5-5.3	4.6	3.7-5.8			2.1	1.7-2.6	2.0	1.7-2.5
Myocytes % of tissue area										
Total	67.9	64.5-71.5	64.0	60.7-67.4	88.6	84.9-92.4	78.3	74.4-82.4	84.1	79.9-88.6
Epicardial	55.4	49.2-62.3	48.6	42.9-55.0			67.7	60.1-76.2	81.4	72.4-91.5
Endocardial	79.8	77.3-82.3	79.3	76.7-82.0			87.4	84.6-90.2	82.2	83.5-88.9

Values are geometric means and corresponding 95% CIs of the predictions.

Abbreviations as in Table 2.

**Central Illustration:**
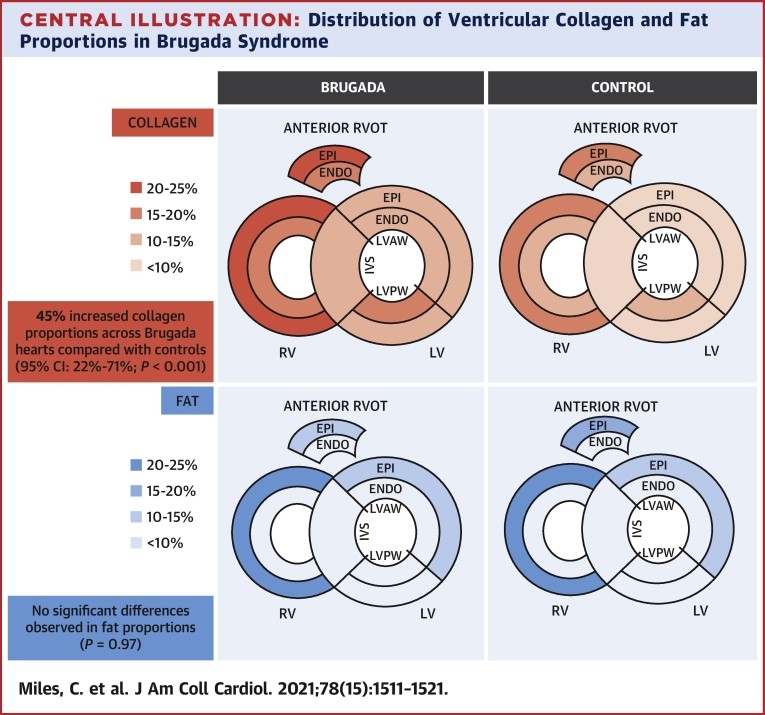
Distribution of Ventricular Collagen and Fat Proportions in Brugada Syndrome Collagen **(top)** and fat **(bottom)** proportions were determined from 6 sampled regions of ventricular myocardium. The highest proportions of collagen were observed within the epicardial RVOT (23.7%; 95% CI: 20.8%-26.9%) and RV (23.0%; 95% CI: 20.3%-26.1%) of the BrS group. Cardiac tissue from BrS decedents demonstrated a higher proportion of collagen when compared with noncardiac death control subjects (ratio 1.45; 95% CI: 1.22-1.71; *P <* 0.001), irrespective of sampling location or myocardial layer. After adjustment for sex and age at death, both BrS and control groups showed similar fat proportions *(P =* 0.97). BrS = Brugada syndrome; ENDO = endocardial; EPI = epicardial; IVS = interventricular septum; LV = left ventricle; LVAW = left ventricular anterolateral wall; LVPW = left ventricular posterior wall; RV = right ventricle; RVOT = right ventricular outflow tract.

Cardiac tissue from BrS decedents demonstrated a higher proportion of collagen when compared with control subjects (ratio 1.45; 95% CI: 1.22-1.71; *P <* 0.001). Between groups, there were no significant interactions with respect to collagen proportion and sampling location (RVOT, RV, IVS, anterior LV, posterior LV) or collagen proportion and tissue layer (epicardium/endocardium). Maximal increases in collagen proportion were observed in the antemortem BrS cohort (n = 6) when compared with control subjects (ratio 1.66; 95% CI: 1.25-2.20; *P =* 0.001). The familial BrS cohort (n = 18) also demonstrated significantly higher collagen proportions (ratio 1.42; 95% CI: 1.17-1.71; *P <* 0.001). However, there was insufficient evidence to support differences in collagen proportion in the pathogenic/likely pathogenic *SCN5A* SADS group (n = 4), when compared with control subjects (ratio 1.29; 95% CI: 0.92-1.81; *P =* 0.14). Similarly, there was insufficient evidence to support differences in collagen proportion in all cases harboring a pathogenic/likely pathogenic *SCN5A* variant (n = 5), when compared with control subjects (ratio 1.23; 95% CI: 0.75-1.43; *P =* 0.27) or genotype negative antemortem/familial BrS cases (n = 13) (ratio 0.87; 95% CI: 0.64-1.18; *P =* 0.37). In multivariable analysis, there were no significant differences in collagen proportion according to sex *(P =* 0.20), age at death *(P =* 0.91), or body mass index (*P* = 0.92).

Overall, the proportion of fat was higher in female than in male hearts (ratio 1.69; 95% CI: 1.21-2.36; *P =* 0.002) and in older compared with younger individuals (ratio per 5-year age increment: 1.08; 95% CI: 1.03-1.15; *P =* 0.004). Maximal differences in fat proportion (women vs men) were in the RVOT (ratio 2.80; 95 CI: 1.65-4.70; *P <* 0.001) and RV (2.02; 95% CI: 1.22-3.40; *P =* 0.006). There was insufficient evidence to support differences in fat proportion according to body mass index *(P =* 0.23). After adjustment for sex and age at death, both BrS and control groups showed similar fat proportions, with no significant differences with respect to sampling location or myocardial layer *(P =* 0.97).

## Discussion

In this study, we demonstrate that BrS is associated with increased collagen content throughout RV and LV myocardium, irrespective of sampling location or myocardial layer. These findings were observed in the absence of overt histological abnormality. To our knowledge, this is the largest study evaluating cardiac tissue from BrS decedents, incorporating blinded automated quantification of myocardial tissue components in a reproducible manner. Moreover, we go beyond previous reports in systematically assessing microstructural abnormalities in tissue architecture within the LV myocardium. Conceivably, we propose that proportional increases in epicardial collagen within the thin-walled RVOT may contribute toward a pathological tissue substrate and discontinuity of conduction in BrS. In the setting of a disturbance in transmural ion channel distribution and reduced sodium current, this would give rise to susceptibility to re-entry and arrhythmogenesis.

### Pathological findings

Overall, we identified a 45% increase in collagen proportions in the BrS group compared with control subjects, with the highest proportion of collagen (24%) found within RVOT epicardium. These data are consistent with previous studies reporting the presence of interstitial fibrosis, fractionated electrograms, and low-voltage regions within RVOT epicardium, and may be in keeping with the association of late potentials on signal-averaged electrocardiography with elevated arrhythmic risk (9,20,21). Taken together, these data ultimately support the depolarization hypothesis underlying conduction delay in BrS. Previously, Nademanee et al (10) evaluated cardiac tissue from a group of 6 postmortem SADS cases with a familial diagnosis of BrS, reporting maximal increases (32%) in collagen proportion in RVOT epicardium with respect to 6 age- and sex-matched control subjects (10). We confirmed an increasing gradient of RV endocardial to epicardial collagen proportion, but did not observe significant intergroup differences between sampling locations. Therefore, in BrS, we hypothesize that normal RV structural heterogeneities are responsible for increased vulnerability to conduction slowing in the presence of diminished sodium current and increased fibrotic burden (22).

Others have also demonstrated the presence of pathological histological substrate in BrS. In 2018, Pieroni et al (23) reported interstitial and replacement fibrosis in RVOT samples from 15 of 20 (75%) BrS patients undergoing RV biopsy. Interestingly, 80% of these patients also showed evidence of myocardial inflammation with lymphomononuclear infiltrates comprised of activated T-lymphocytes. Although we did not find overt features of inflammatory infiltrates in postmortem tissue samples from either BrS or control subjects, it is important to note that biopsies in the study performed by Pieroni et al (23) were performed under guidance from 3-dimensional electroanatomic mapping and included immunohistochemical analysis. The difference in sampling technique and use of autopsy samples in our SCD cohort cannot exclude temporal differences in disease pathogenesis or localized regions of myocardial inflammation elsewhere in BrS myocardium. However, our observations were broadly in line with a pathological report by Zumhagen et al (24), which did not describe inflammatory changes in myocardial biopsy samples from 21 patients with BrS.

In the present study, we showed that there were no significant differences in myocardial fat proportion between BrS case and control subjects. A similar conclusion was reached by Nademanee et al (10), which is in contrast to earlier reports suggesting that myocardial fatty infiltration in ACM may overlap histologically with BrS (25,26). However, our data did yield age- and sex-specific differences in fat proportion, with increased fat content associated with older age and female sex demonstrating a 2-fold increase in RV fat proportion when compared with men. This is consistent with other pathological studies demonstrating similar increases in cardiac fatty tissue deposition, particularly among elderly women (27,28).

### Clinical correlations

Maximal differences in collagen proportion were observed in the antemortem BrS subgroup (n = 6), which showed a 66% increase when compared with control subjects. All antemortem BrS decedents had achieved a probable or definite diagnosis of BrS by the Shanghai criteria (score >3.5), most having demonstrated a spontaneous type 1 ECG during life, and one having tested positive for a pathogenic *SCN5A* mutation. Although the increased collagen proportions in this group may associate with a higher diagnostic certainty for BrS, there were no significant differences in collagen proportion between antemortem and familial BrS groups. However, our data did not yield any significant differences in collagen proportion between SADS decedents with pathogenic or likely pathogenic *SCN5A* variants (n = 4) and control subjects. This small group consisted of 3 missense mutations and 1 in-frame deletion, all deemed pathogenic or likely pathogenic as per ACMG criteria, and 2 of the variants had previously been reported in probands with both BrS and LQTS type 3. From this standpoint, the small sample size, lack of an antemortem or familial discernible phenotype, and pleotropic effects of *SCN5A* variants may partly explain the lack of a statistically significant finding of greater fibrosis in this subgroup.

### Underlying mechanisms and LV involvement

Despite ongoing debate about whether the repolarization or depolarization hypothesis predominates in BrS, there is growing consensus that the syndrome is associated with mild structural abnormalities and a reduced RVOT conduction reserve has been postulated (22,29). Importantly, our data support that these are independent of *SCN5A* genotype. We speculate that an elevated fibrotic substrate burden in BrS may affect heightened arrhythmogenesis through current-to-load mismatch and excitation failure (30). Furthermore, our data cast new light on the pathological substrate underlying BrS, specifically finding a consistent increase in collagen proportion throughout ventricular myocardium, not just the RVOT. Indeed, widespread proportionate increases in collagen deposition suggests disease involvement throughout both ventricles and may lend support to activation of molecular pathways implicated in myocardial fibrosis. Here, transcriptomic studies utilizing RNA extracted from ventricular myocardium may facilitate assessment of global and/or regional differences in molecular pathways implicated in collagen synthesis in addition to the impact of rare *SCN5A* variants on collagen expression.

Others have shown the presence of LV fibrosis in BrS, both in vivo in a few cases using CMR and during histological examination (10,11). Experimental models also provide a mechanistic basis for this association, albeit in hearts from a haploinsufficient *SCN5A*^+/−^ mouse model. For example, Jeevaratnam et al (31) reported fibrotic changes within both right and left ventricles, where epicardial activation analysis also demonstrated increased late conducting components. Although CMR late gadolinium enhancement imaging is an established technique to assess replacement patterns of myocardial fibrosis, its sensitivity is limited for the detection of diffuse interstitial fibrosis (32). The findings from this study warrant further investigation of BrS patients in vivo using contemporary CMR techniques such as T_1_ mapping and extracellular volume assessment, as described elsewhere (33).

### Study limitations

First, although a case-control study design is recommended for rare outcomes, there are some caveats that invite caution. Our study was retrospective in nature and BrS is of low prevalence in the general population. Therefore, we were limited by recruitment of SCD cases over a prolonged period. Second, for SADS cases with a familial diagnosis of BrS, recruitment relied upon review of investigations performed at our hospital. It is possible that additional SADS cases from the same overall cohort may have been eligible for inclusion if clinical data were available from family screening conducted at other centers. Moreover, although the diagnosis of familial BrS was achieved by accepted international criteria (2), we cannot exclude the potential for misclassification bias among the familial BrS group in absence of an antemortem diagnosis. However, we consider this unlikely given the inherited nature of BrS, lack of alternative phenotype within the family, and autopsy-negative sudden death in the proband. Third, this study relied upon SCD referral to an expert national cardiac pathology center. This may introduce ascertainment bias, because the incidence of SCD in BrS is low and such cases may represent a more severe phenotype where myocardial fibrosis is more prevalent. Finally, postmortem genetic testing was limited by the number of retained genomic tissue samples—a higher genetic yield would improve our ability to investigate potential associations between genotype and fibrotic burden.

## Conclusions

In this study, we systematically demonstrate that BrS is associated with increased collagen content throughout both ventricles, with maximal collagen proportions observed within BrS RVOT epicardium. These findings were not apparent during macroscopic inspection of the heart or conventional histological examination. Our study emphasizes the spectrum of normal variation of collagen and fat content within both ventricles, in contrast to a pathological increase in collagen content found in BrS. In addition, we highlight the potential utility of automated digital pathology software to complement expertise provided by the pathologist. Further research is required to elucidate the mechanistic basis of fibrosis in BrS and understand its cumulative effect on arrhythmic risk.

Perspectives **COMPETENCY IN MEDICAL KNOWLEDGE:** BrS is associated with higher proportionate collagen content throughout both LV and RV myocardium.

**TRANSLATIONAL OUTLOOK:** Future studies should incorporate mRNA sequencing of myocardial tissue specimens for transcriptome-wide analysis of molecular pathways involved in collagen synthesis in patients with BrS.

## Funding Support and Author Disclosures

Dr Miles is the recipient of a British Heart Foundation Clinical Research Training Fellowship (FS/18/28/33549). Drs Miles, Ensam, and Behr have received research funding from the Robert Lancaster Memorial Fund, sponsored by McColl’s Retail Group Ltd, United Kingdom. Drs Papadakis, Finocchiaro, Ensam, Basu, Parry-Williams, and MacLachlan have received research fellowship grants from Cardiac Risk in the Young (CRY), United Kingdom. Dr Gray is the recipient of a National Health and Medical Research Council Early Career Fellowship (Fellowship #1122330). All other authors have reported that they have no relationships relevant to the contents of this paper to disclose.
