# Supplementary material for: Biventricular Myocardial Fibrosis and Sudden Death in Patients With Brugada Syndrome
Source: J Am Coll Cardiol. 2021 Oct 12;78(15):1511–21. doi: 10.1016/j.jacc.2021.08.010 (PMC8504200; doi:10.1016/j.jacc.2021.08.010)
Supplement: Supplemental Tables 1 and 2 [file mmc1.docx]

# Supplemental Table 1: Post-Mortem Genetic Testing

| **Gene** | **Gene name** |
| --- | --- |
|  |  |
| ***Inherited Arrhythmias*** | |
| *Long QT syndrome - 16 genes* | |
| *KCNQ1* | potassium voltage-gated channel, KQT-like subfamily, member 1 |
| *KCNH2* | potassium voltage-gated channel, subfamily H (eag-related), member 2 |
| *SCN5A* | sodium channel, voltage-gated, type V, alpha subunit |
| *ANK2* | ankyrin 2, neuronal |
| *KCNE1* | potassium voltage-gated channel, Isk-related family, member 1 |
| *KCNE2* | potassium voltage-gated channel, Isk-related family, member 2 |
| *KCNJ2* | potassium inwardly-rectifying channel, subfamily J, member 2 |
| *CACNA1C* | calcium channel, voltage-dependent, L type, alpha 1C subunit |
| *CAV3* | caveolin 3 |
| *SCN4B* | sodium channel, voltage-gated, type IV, beta |
| *AKAP9* | A kinase (PRKA) anchor protein (yotiao) 9 |
| *SNTA1* | syntrophin, alpha 1 (dystrophin-associated protein A1, 59kDa, acidic component) |
| *KCNJ5* | potassium inwardly-rectifying channel, subfamily J, member 5 |
| *RYR2* | ryanodine receptor 2 (cardiac) |
| *KCNE3* | potassium voltage-gated channel, Isk-related family, member 3 |
| *CALM1* | calmodulin 1 (phosphorylase kinase, delta) |
| *Short QT syndrome - 4 genes* | |
| *KCNH2* | potassium voltage-gated channel, subfamily H (eag-related), member 2 |
| *KCNQ1* | potassium voltage-gated channel, KQT-like subfamily, member 1 |
| *KCNJ2* | potassium inwardly-rectifying channel, subfamily J, member 2 |
| *CACNA2D1* | calcium channel, voltage-dependent, alpha 2/delta subunit 1 |
|  |  |
| *Brugada syndrome - 14 genes* | |
| *SCN5A* | sodium channel, voltage-gated, type V, alpha subunit |
| *GPD1L* | glycerol-3-phosphate dehydrogenase 1-like |
| *CACNA1C* | calcium channel, voltage-dependent, L type, alpha 1C subunit |
| *CACNB2* | calcium channel, voltage-dependent, beta 2 subunit |
| *KCNE3* | potassium voltage-gated channel, Isk-related family, member 3 |
| *SCN3B* | sodium channel, voltage-gated, type III, beta |
| *KCNJ8* | potassium inwardly-rectifying channel, subfamily J, member 8 |
| *CACNA2D1* | calcium channel, voltage-dependent, alpha 2/delta subunit 1 |
| *RANGRF* | RAN guanine nucleotide release factor |
| *HCN4* | hyperpolarization activated cyclic nucleotide-gated potassium channel 4 |
| *KCNH2* | potassium voltage-gated channel, subfamily H (eag-related), member 2 |
| *PKP2* | plakophilin 2 |
| *ABCC9* | ATP-binding cassette, sub-family C (CFTR/MRP), member 9 |
| *SCN1B* | sodium channel, voltage-gated, type i, beta subunit |
|  |  |
| *Catecholaminergic polymorphic ventricular tachycardia - 5 genes* | |
| *RYR2* | ryanodine receptor 2 (cardiac) |
| *CASQ2* | calsequestrin 2 (cardiac muscle) |
| *KCNE1* | potassium voltage-gated channel, Isk-related family, member 1 |
| *KCNJ2* | potassium inwardly-rectifying channel, subfamily J, member 2 |
| *CALM1* | calmodulin 1 (phosphorylase kinase, delta) |
|  |  |
| ***Cardiomyopathies*** | |
| *Hypertrophic cardiomyopathy - 35 genes* | |
| *MYBPC3* | myosin binding protein C, cardiac |
| *MYH7* | myosin, heavy chain 7, cardiac muscle, beta |
| *TNNT2* | troponin T type 2 (cardiac) |
| *TNNI3* | troponin I type 3 (cardiac) |
| *TPM1* | tropomyosin 1 (alpha) |
| *MYL3* | myosin, light chain 3, alkali; ventricular, skeletal, slow |
| *MYL2* | myosin, light chain 2, regulatory, cardiac, slow |
| *ACTC1* | actin, alpha, cardiac muscle 1 |
| *CSRP3* | cysteine and glycine-rich protein 3 (cardiac LIM protein) |
| *PRKAG2* | protein kinase, AMP-activated, gamma 2 non-catalytic subunit |
| *MYPN* | myopalladin |
| *TTN* | titin |
| *TCAP* | titin-cap (telethonin) |
| *TNNC1* | troponin C type 1 (slow) |
| *JPH2* | junctophilin 2 |
| *ACTN2* | actinin, alpha 2 |
| *MYH6* | myosin, heavy chain 6, cardiac muscle, alpha |
| *MYLK2* | myosin light chain kinase 2 |
| *ANKRD1* | ankyrin repeat domain 1 (cardiac muscle) |
| *VCL* | vinculin |
| *CALR3* | calreticulin 3 |
| *MYOZ2* | myozenin 2 |
| *NEXN* | nexilin (F actin binding protein) |
| *RAF1* | v-raf-1 murine leukemia viral oncogene homolog 1 |
| *CACNA1C* | calcium channel, voltage-dependent, L type, alpha 1C subunit |
| *CAV3* | caveolin 3 |
| *CASQ2* | calsequestrin 2 (cardiac muscle) |
| *DES* | desmin |
| *FXN* | frataxin |
| *GLA* | galactosidase, alpha |
| *PDLIM3* | PDZ and LIM domain 3 |
| *LAMP2* | lysosomal-associated membrane protein 2 |
| *PTPN11* | protein tyrosine phosphatase, non-receptor type 11 |
| *CRYAB* | crystallin, alpha B |
| *KCNQ1* | potassium voltage-gated channel, KQT-like subfamily, member 1 |
|  |  |
| *Dilated cardiomyopathy - 40 genes* | |
| *LMNA* | lamin A/C |
| *MYH7* | myosin, heavy chain 7, cardiac muscle, beta |
| *TTN* | titin |
| *DSP* | desmoplakin |
| *MYBPC3* | myosin binding protein C, cardiac |
| *TNNT2* | troponin T type 2 (cardiac) |
| *SCN5A* | sodium channel, voltage-gated, type V, alpha subunit |
| *RBM20* | RNA binding motif protein 20 |
| *DMD* | dystrophin |
| *TPM1* | tropomyosin 1 (alpha) |
| *LDB3* | LIM domain binding 3 |
| *DES* | desmin |
| *TNNI3* | troponin I type 3 (cardiac) |
| *MYPN* | myopalladin |
| *MYH6* | myosin, heavy chain 6, cardiac muscle, alpha |
| *TNNC1* | troponin C type 1 (slow) |
| *ANKRD1* | ankyrin repeat domain 1 (cardiac muscle) |
| *TCAP* | titin-cap (telethonin) |
| *VCL* | vinculin |
| *PLN* | phospholamban |
| *ACTC1* | actin, alpha, cardiac muscle 1 |
| *ACTN2* | actinin, alpha 2 |
| *DSG2* | desmoglein 2 |
| *CSRP3* | cysteine and glycine-rich protein 3 (cardiac LIM protein) |
| *FKTN* | fukutin |
| *SGCD* | sarcoglycan, delta (35kDa dystrophin-associated glycoprotein) |
| *TAZ* | tafazzin |
| *CRYAB* | crystallin, alpha B |
| *NEXN* | nexilin (F actin binding protein) |
| *PKP2* | plakophilin 2 |
| *ABCC9* | ATP-binding cassette, sub-family C (CFTR/MRP), member 9 |
| *LAMA4* | laminin, alpha 4 |
| *DSC2* | desmocollin 2 |
| *TMPO* | thymopoietin |
| *LAMP2* | lysosomal-associated membrane protein 2 |
| *HFE* | HFE |
| *PDLIM3* | PDZ and LIM domain 3 |
| *JUP* | junction plakoglobin |
| *FKRP* | fukutin-related protein |
| *SDHA* | flavoprotein subunit of succinate dehydrogenase |
|  |  |
| *Arrhythmogenic cardiomyopathy - 12 genes* | |
| *PKP2* | plakophilin 2 |
| *DSP* | desmoplakin |
| *DSG2* | desmoglein 2 |
| *DSC2* | desmocollin 2 |
| *JUP* | junction plakoglobin |
| *TTN* | titin |
| *TMEM43* | transmembrane protein 43 |
| *RYR2* | ryanodine receptor 2 (cardiac) |
| *DES* | desmin |
| *TGFB3* | transforming growth factor, beta 3 |
| *LMNA* | lamin A/C |
| *SCN5A* | sodium channel, voltage-gated, type V, alpha subunit |
|  |  |
| *Restrictive cardiomyopathy (RCM) - 9 genes* | |
| *TNNI3* | troponin I type 3 (cardiac) |
| *DES* | desmin |
| *MYH7* | myosin, heavy chain 7, cardiac muscle, beta |
| *TNNT2* | troponin T type 2 (cardiac) |
| *ACTC1* | actin, alpha, cardiac muscle 1 |
| *MYL3* | myosin, light chain 3, alkali; ventricular, skeletal, slow |
| *MYL2* | myosin, light chain 2, regulatory, cardiac, slow |
| *TPM1* | tropomyosin 1 (alpha) |
| *MYPN* | myopalladin |
|  |  |
| *Left ventricular non-compaction (LVNC) - 7 genes* | |
| *MYH7* | myosin, heavy chain 7, cardiac muscle, beta |
| *MYBPC3* | myosin binding protein C, cardiac |
| *TAZ* | tafazzin |
| *TPM1* | tropomyosin 1 (alpha) |
| *ACTC1* | actin, alpha, cardiac muscle 1 |
| *TNNT2* | troponin T type 2 (cardiac) |
| *CASQ2* | calsequestrin 2 (cardiac muscle) |

Adapted from: (1)

1. Lahrouchi N, Raju H, Lodder EM, et al. Utility of Post-Mortem Genetic Testing in Cases of Sudden Arrhythmic Death Syndrome. J. Am. Coll. Cardiol. 2017;69:2134–2145.

**Supplemental Table 2: Overview of Collagen Proportions Stratified by Myocardial Region and Layer**

| CaseID | RVOT |  |  | RV |  |  | Septum | Anterior LV | |  | Posterior LV | |  |
| --- | --- | --- | --- | --- | --- | --- | --- | --- | --- | --- | --- | --- | --- |
|  | Total | Epi | Endo | Total | Epi | Endo | Total | Total | Epi | Endo | Total | Epi | Endo |
| BrSA1 | 23.6 | 22.5 | 24.6 | 33.2 | 30.0 | 36.3 | 15.0 | 16.0 | 14.0 | 18.3 | 16.5 | 13.0 | 19.2 |
| BrSA2 | 18.0 | 20.8 | 15.5 | 15.8 | 17.8 | 14.3 | 9.6 | 7.1 | 6.6 | 7.6 | 8.4 | 8.0 | 8.8 |
| BrSA3 | 23.0 | 29.0 | 19.3 | 15.1 | 19.5 | 10.0 | 13.0 | 12.0 | 9.2 | 14.3 | 16.3 | 13.5 | 18.2 |
| BrSA4 | 37.1 | 38.4 | 35.3 | 30.0 | 30.6 | 28.1 | 28.6 | 23.9 | 23.3 | 24.2 | 24.6 | 25.2 | 24.2 |
| BrSA5 | 12.6 | 12.3 | 12.9 | 23.1 | 25.2 | 20.0 | 23.3 | 11.8 | 11.9 | 11.0 | 22.0 | 15.3 | 29.6 |
| BrSA6 | 24.0 | 30.0 | 17.5 | 23.7 | 25.3 | 22.0 | 20.2 | 19.7 | 17.7 | 21.0 | 12.6 | 10.9 | 14.0 |
| BrSF7 | 28.8 | 28.3 | 29.2 | 22.0 | 19.8 | 23.8 | 33.2 | 22.6 | 15.9 | 29.2 | 35.9 | 25.6 | 50.5 |
| BrSF8 |  |  |  | 20.0 | 21.8 | 17.0 | 9.7 | 15.7 | 15.7 | 15.7 | 13.7 | 13.5 | 15.1 |
| BrSF9 | 34.5 | 36.1 | 32.7 | 27.7 | 31.5 | 25.2 | 18.3 | 16.9 | 15.7 | 18.2 | 23.2 | 22.1 | 24.2 |
| BrSF10 | 19.2 | 19.7 | 18.0 | 20.7 | 20.2 | 20.4 | 10.8 | 14.4 | 15.8 | 11.5 | 13.7 | 10.6 | 16.2 |
| BrSF11 | 23.2 | 20.1 | 27.1 | 17.0 | 16.9 | 16.2 | 10.4 | 10.7 | 10.3 | 11.3 | 13.5 | 10.7 | 15.7 |
| BrSF12 | 20.4 | 27.9 | 15.7 | 15.2 | 21.3 | 9.8 | 7.3 | 7.6 | 7.0 | 8.2 | 12.6 | 9.9 | 14.4 |
| BrSF13 | 22.5 | 31.8 | 13.3 | 21.1 | 38.2 | 11.4 | 7.1 | 8.3 | 10.1 | 10.1 | 16.2 | 21.9 | 10.8 |
| BrSF14 | 36.4 | 38.6 | 33.1 | 32.4 | 36.9 | 27.7 | 20.3 | 15.4 | 13.9 | 12.1 | 18.4 | 16.8 | 19.7 |
| BrSF15 | 24.4 | 25.4 | 23.0 | 26.7 | 29.1 | 23.9 | 13.0 |  |  |  | 14.9 | 13.2 | 16.6 |
| BrSF16 | 21.8 | 25.0 | 18.4 | 15.9 | 13.8 | 17.1 | 18.3 | 14.7 | 13.4 | 15.7 | 16.5 | 14.4 | 18.8 |
| BrSF17 | 13.6 | 15.6 | 16.5 | 14.3 | 13.4 | 15.1 | 5.8 | 10.0 | 10.9 | 8.9 | 8.2 | 8.2 | 7.8 |
| BrSF18 | 17.1 | 21.3 | 12.9 | 11.7 | 12.3 | 10.2 | 8.3 | 9.5 | 12.8 | 7.7 | 7.7 | 7.2 | 7.8 |
| BrSF19 | 18.9 | 19.0 | 18.0 | 21.0 | 21.3 | 19.5 | 9.8 | 10.6 | 9.3 | 11.7 | 9.8 | 9.3 | 10.2 |
| BrSF20 | 27.2 | 30.6 | 24.7 | 25.1 | 26.8 | 23.2 | 20.1 | 20.4 | 22.4 | 17.6 |  |  |  |
| BrSF21 | 31.7 | 35.9 | 26.5 | 31.2 | 37.6 | 25.4 | 20.2 | 12.6 | 12.8 | 12.4 | 18.0 | 20.4 | 16.9 |
| BrSF22 | 17.8 | 24.8 | 12.9 | 21.1 | 28.7 | 13.9 | 13.8 | 7.9 | 7.9 | 7.9 | 10.5 | 12.1 | 10.0 |
| BrSF23 | 22.0 | 26.5 | 17.0 | 21.9 | 28.2 | 16.1 | 8.7 | 11.9 | 12.2 | 10.4 | 9.4 | 8.5 | 10.1 |
| BrSF24 | 21.5 | 23.0 | 19.6 | 23.4 | 24.0 | 20.1 | 9.5 | 12.9 | 12.5 | 13.3 | 12.3 | 8.9 | 16.2 |
| BrSG25 | 15.5 | 18.5 | 12.2 | 20.3 | 28.2 | 14.6 | 9.0 | 8.0 | 16.9 | 4.5 | 8.5 | 6.7 | 10.0 |
| BrSG26 | 25.2 | 27.4 | 22.4 | 16.0 | 14.9 | 13.3 | 11.9 | 11.6 | 15.1 | 9.1 | 15.1 | 12.5 | 17.2 |
| BrSG27 | 21.4 | 21.9 | 19.9 | 21.9 | 26.4 | 18.7 | 11.2 | 11.4 | 14.5 | 8.4 | 15.6 | 13.6 | 16.1 |
| BrSG28 | 10.3 | 6.2 | 14.5 | 18.9 | 16.2 | 22.5 | 16.9 | 16.5 | 14.4 | 18.3 | 21.0 | 19.4 | 22.3 |
| C1 | 18.6 | 20.4 | 17.0 | 16.4 | 18.0 | 14.6 | 12.9 | 14.2 | 12.8 | 15.4 | 9.8 | 8.3 | 11.9 |
| C2 | 16.0 | 18.2 | 13.3 | 14.6 | 13.9 | 15.3 | 12.1 | 7.0 | 7.3 | 6.6 | 7.6 | 7.0 | 7.3 |
| C3 | 11.1 | 12.6 | 9.5 | 14.4 | 18.8 | 11.2 | 7.2 | 5.5 | 5.3 | 5.1 | 9.0 | 9.0 | 7.7 |
| C4 | 19.1 | 21.4 | 16.8 | 21.9 | 30.7 | 17.2 | 10.0 | 10.6 | 11.1 | 10.1 | 9.7 | 9.4 | 9.9 |
| C5 | 12.1 | 13.5 | 9.9 | 15.7 | 13.4 | 17.5 | 7.9 | 9.5 | 10.6 | 8.1 | 17.9 | 17.0 | 19.6 |
| C6 | 22.6 | 26.7 | 16.9 | 15.8 | 16.3 | 14.0 | 8.8 | 9.5 | 10.8 | 7.8 | 14.0 | 10.7 | 16.8 |
| C7 | 22.3 | 23.6 | 19.8 |  |  |  | 5.5 | 7.3 | 7.9 | 5.4 | 7.5 | 5.3 | 9.1 |
| C8 | 13.6 | 15.4 | 12.0 | 14.1 | 20.3 | 8.7 | 8.5 | 9.7 | 12.0 | 9.2 | 10.2 | 8.7 | 11.7 |
| C9 | 29.4 | 32.9 | 25.4 | 23.7 | 19.6 | 27.4 | 20.6 | 22.6 | 17.7 | 30.0 | 22.3 | 21.4 | 26.0 |
| C10 | 17.2 | 19.6 | 14.6 | 19.8 | 21.4 | 18.4 | 6.3 | 9.6 | 13.4 | 7.2 | 7.6 | 7.4 | 7.5 |
| C11 | 11.2 | 14.5 | 7.7 | 9.4 | 10.4 | 8.1 | 4.3 | 5.7 | 7.0 | 4.6 | 6.7 | 8.0 | 5.7 |
| C12 | 12.7 | 14.1 | 11.3 | 6.7 | 7.0 | 6.0 | 4.0 | 6.3 | 7.3 | 5.8 | 6.1 | 5.9 | 6.2 |
| C13 | 27.3 | 29.9 | 24.0 | 18.7 | 23.6 | 15.1 | 12.9 | 14.9 | 17.6 | 11.8 | 17.7 | 20.0 | 15.0 |
| C14 | 19.4 | 22.0 | 16.3 | 21.1 | 19.6 | 21.7 | 16.0 | 21.8 | 20.2 | 23.5 | 15.6 | 17.6 | 15.0 |
| C15 | 6.6 | 4.4 | 8.8 | 10.9 | 10.2 | 11.3 | 5.4 | 6.2 | 5.1 | 7.4 | 8.5 | 8.2 | 9.2 |
| C16 | 12.2 | 11.7 | 12.9 | 14.7 | 16.7 | 12.4 | 5.6 | 5.7 | 6.3 | 5.6 | 4.3 | 4.2 | 5.5 |
| C17 | 13.2 | 10.8 | 15.8 | 12.5 | 13.9 | 9.8 | 6.4 | 6.0 | 5.0 | 7.2 | 10.2 | 6.5 | 6.8 |
| C18 | 18.6 | 20.2 | 14.9 | 19.2 | 19.7 | 18.5 | 12.4 | 8.7 | 7.7 | 9.3 | 13.1 | 13.2 | 12.4 |
| C19 | 7.1 | 9.1 | 6.8 | 12.3 | 13.6 | 10.9 | 8.0 | 8.1 | 10.3 | 5.0 | 11.3 | 11.1 | 11.4 |
| C20 | 39.8 | 46.1 | 32.2 | 34.1 | 35.6 | 30.9 | 13.6 | 14.3 | 15.5 | 12.4 | 14.8 | 12.7 | 17.0 |
| C21 | 12.3 | 13.8 | 10.5 | 16.7 | 16.5 | 13.8 | 8.9 | 5.8 |  |  | 11.9 | 7.4 | 14.6 |
| C22 | 12.1 | 10.7 | 13.4 | 14.1 | 11.8 | 16.4 | 15.7 | 15.6 | 11.2 | 20.3 | 13.5 | 11.6 | 15.3 |
| C23 |  |  |  | 24.1 |  |  | 11.4 | 18.9 | 19.3 | 14.3 | 14.0 | 13.2 | 13.3 |
| C24 | 4.7 | 5.4 | 3.9 | 16.0 | 20.6 | 13.7 | 9.9 | 10.3 | 9.7 | 10.5 | 11.0 | 10.8 | 11.1 |
| C25 | 11.8 | 13.9 | 9.6 | 10.9 | 11.0 | 10.4 | 8.6 | 5.6 | 5.3 | 5.8 | 4.6 | 2.1 | 6.0 |
| C26 | 21.5 | 24.6 | 17.1 | 22.1 | 29.6 | 15.7 | 8.5 | 8.5 | 9.0 | 7.6 | 8.5 | 9.9 | 7.3 |
| C27 | 30.3 | 32.4 | 26.8 | 20.7 | 20.2 | 20.1 | 9.0 | 11.1 | 11.2 | 10.4 | 11.5 | 10.3 | 12.6 |
| C28 | 5.3 | 3.9 | 7.1 | 15.4 |  |  | 3.9 | 3.7 | 3.5 | 3.3 | 3.7 | 2.3 | 6.3 |
| C29 | 8.4 | 5.9 | 11.3 |  |  |  | 9.6 | 8.3 | 8.5 | 8.3 | 9.2 | 8.9 | 8.9 |

Raw data at original scale (%) are shown. For the purpose of analyses, logarithmic scales were used in order to satisfy the modeling assumptions (see statistical methods).
